# Emotional eating and disordered eating behaviors in children and adolescents with type 1 diabetes

**DOI:** 10.1038/s41598-022-26271-2

**Published:** 2022-12-17

**Authors:** Carlo Ripoli, Maria Rossella Ricciardi, Ester Zuncheddu, Maria Rosaria Angelo, Anna Paola Pinna, Daniela Ripoli

**Affiliations:** 1 Pediatric Diabetology Unit, ASL Cagliari, Sardinia, Italy; 2 Pediatric Emergency Unit, Arnas G. Brotzu Cagliari, Sardinia, Italy; 3 Quartu Sant’Elena (Cagliari), Sardinia, Italy

**Keywords:** Type 1 diabetes, Human behaviour

## Abstract

Disordered eating behaviors (DEB) are more common in adolescents with type 1 diabetes (T1D) than in peers without diabetes. Emotional eating is a risk factor for binge eating in children and adolescents in the general population and is associated with increased intake of high energy-dense foods rich in sugars and fats. The primary objective is to evaluate whether emotional eating is associated with the metabolic control (glycated hemoglobin, plasma lipids and uric acid) in children and adolescents with type 1 diabetes and whether subjects with DEB (DEPS-R ≥ 20) have higher emotional eating than those without DEB. The secondary objective is to evaluate whether emotional eating is associated with the different symptoms of DEB. Emotional eating is positively correlated with HbA1c, total and LDL cholesterol values in children and adolescents with T1D. Subjects with DEB have a higher emotional eating score than subjects without DEB. Disinhibition is the most common disordered eating behavior in children and adolescents with T1D and is associated with a higher emotional eating score. Early identification and treatment of emotional eating could be tools for preventing DEB in people with type 1 diabetes. A total of 212 adolescents with T1D completed two self-administered questionnaires: the Diabetes Eating Problem Survey-Revised (DEPS-R) and the Emotional Eating Scale for Children and Adolescents (EES-C). Demographic (age, sex, duration of the disease), anthropometric (weight, height, BMI, BMI-SDS), therapeutic (type of insulin therapy, daily insulin dose) and metabolic (HbA1c, total cholesterol, HDL, LDL, triglycerides, uric acid) data were taken from the patients' medical records. The presence of other autoimmune diseases was also recorded.

## Introduction

Type 1 diabetes mellitus (T1D) is a chronic disease that requires lifelong insulin replacement therapy. This can be performed with multiple daily injections or with the use of an insulin pump and must be combined with blood glucose monitoring and quantification of carbohydrate intake at meals to establish the correct doses of insulin. This has a major impact on the quality of life of children with diabetes and their families and can lead to diabetes-related stress^1^. A recent national study in Sweden found that children and young people with T1D have more than twice the risk of psychiatric illness than their peers and siblings without diabetes^2^. Eating disorders (ED), after substance abuse, are the most frequent psychiatric disorders in adolescents with T1D and, as in the general population, predominantly affect females^2,3^.

*Disordered eating behaviors* (DEB) is a term that encompasses the whole spectrum of pathological behaviors related to eating, i.e., food restriction, excessive exercise to control body weight, binge eating, self-induced vomiting, and use of diuretics and laxatives. In DEB, these pathological behaviors often occur with reduced frequency and intensity that do not allow a diagnosis of ED^4^. Young people with diabetes also have another unique way of controlling body weight, which is the voluntary reduction/omission of insulin therapy, to induce hyperglycemia, glycosuria, ketonuria and weight loss^5–7^. DEB is also more common in young people with T1D than in peers without diabetes^4,8,9^. A recent Italian study performed in 690 adolescents with T1D aged 11–19 found a prevalence of DEB of 28.1% (21% in boys and 35% in girls). Teens with DEB were associated with higher HbA1c, BMI, and emotional and behavioral problems compared to adolescents without DEB. Furthermore, 39% of patients reduced/omitted insulin therapy and had higher glycated hemoglobin values than subjects who did not manipulate the therapy^10^. In some studies, the presence of DEB was also associated with alterations in plasma lipids^11^. These data are consistent with those of several other studies^12–14^. Binge eating is one of the most frequent DEB in screenings performed in adolescents with T1D (30%)^15^. A study performed in 506 Danish adolescents showed that 8.4% had overeating, 18% subclinical binge eating and 7.9% clinical binge eating. Patients with clinical binge eating (defined as ≥ 4 binge eating episodes over the past 28 days) had significantly higher HbA1c values than subjects without overeating^16^.

It is therefore very important to identify individuals at risk of developing DEB at an early stage. In 2010 Markovitz et al. proposed a screening questionnaire for DEB specific for people with diabetes, the Diabetes Eating Problem Survey—Revised (DEPS-R)^17^. This test, consisting of 16 items, allows the identification of individuals at higher risk of developing DEB who should be referred for psychiatric evaluation. The psychometric properties of the DEPS-R were subsequently confirmed by Wisting et al.^18^. Recently Calcaterra et al., starting from a clinimetric evaluation, proposed a division of the 16 items of the DEPS-R into four factors: *restriction and body dissatisfaction, disinhibition, compensatory behaviors, and diabetes management*, which are better suited than the original factors to the symptoms of DEB^19,20^.

Emotional eating is an eating pattern that consists of using food in response to negative emotions such as anxiety, sadness, loneliness, anger, and depression. It is frequent in children and adolescents of the general population and is considered a risk factor for binge eating^21^. It is associated with a dietary pattern characterized by the intake of hyperpalatable foods rich in sugars and fats^22–24^. In 2007 Tanofsky-Kraff modified the Emotional Eating Scale for adults (EES)^25^ adapting it to use in children (Emotional Eating Scale adapted for use in children and adolescents, EES-C). This scale has good convergent and discriminating validity and adequate reliability in the test retest and is considered a suitable tool for assessing emotional eating in children and adolescents between 8 and 18 years^26^.

Currently, the role of *emotional eating* as a risk factor for DEB in children and adolescents with T1D has not been thoroughly investigated. A survey of a small number of patients showed that adolescents with T1D have a higher frequency of emotional eating than their peers and that this correlates positively with HbA1c values^27^. The identification and treatment of emotional eating in adolescents with T1D could be the subject of intervention for the prevention of DEB.

The primary objectives of this work are (a) to assess whether in children and adolescents with T1D the presence of emotional eating is associated with metabolic control (glycated hemoglobin, plasma lipids, uric acid), with demographic (age, sex, duration of diabetes), anthropometric (BMI-SDS), therapeutic (insulin delivery method, with pump or multiple daily injections, and total daily dose), and clinical variables (association with other autoimmune diseases) and (b) whether subjects with DEB have a higher emotional eating score than subjects who do not have DEB. The secondary objective is to evaluate whether emotional eating is associated with the different symptoms (factors) of DEB: *disinhibition, compensatory behaviors, restriction and body dissatisfaction*, as defined in the clinimetric evaluation of the work by Calcaterra et al. and the impact of each of these symptoms on metabolic control (glycated hemoglobin, plasma lipids, uric acid). Disinhibition is the main symptom of binge eating disorder and bulimia nervosa, compensatory behaviors of bulimia nervosa and anorexia nervosa binge/purge subtype. Finally, restriction and body dissatisfaction is the main symptom of anorexia nervosa.

Since the EES-C is validated in several languages but not in Italian, we have validated the Italian version of the scale^28–30^.

## Results

### Characteristics of participants

In this cross-sectional study, 216 patients with T1D were recruited, representing 70% of the subjects aged between 10 and 17 years followed in our centre. Four patients (2 males and 2 females) were excluded because their questionnaires were incomplete. The final sample consisted of 212 patients (59% males) with a mean age of 14.4 ± 2.1 years. Twenty-nine percent of patients were receiving insulin therapy using a pump and the remainder received multiple daily injections. The mean BMI-SDS was −0.26 (SD 0.99), and the median HbA1c value was 7% (IQR 6.6–7.9%). According to the literature data, females have higher HbA1c values than males^31^. Total cholesterol, LDL, and HDL were also higher, while uric values were lower. Patient characteristics are shown in Table 1.

**Table 1:** Patients characteristics.

Variables	All	Males	Females	p-value*
Number %	212	126 (59.4)	86 (40.6)	
Age years	14.4 ± 2.0	14.7 ± 2.0	14.0 ± 2.1	0.156
Diabetes duration years	6.4 ± 4.0	6.6 ± 3.9	6.2 ± 4.2	0.573
BMI-SDS	−0.26 ± 0.99	−0.32 ± 0.89	−0.17 ± 1.12	0.364
**Insulin treatment %**				
Multiple daily injections	70.8	70.9	70.5	0.959
Pump	29.2	29.1	29.5	
Insulin dose U/kg/die	1.0 ± 0.2	1.0 ± 0.2	0.9 ± 0.2	0.128
HbA1c median (IQR) %	7 (6.6, 7.9)	6.8 (6.5, 7.5)	7.3 (6.7, 8.2)	0.003**
mmol/mol	53 (49, 63)	51 (48, 58)	56 (50, 66)	
Total cholesterol mmol/L	4.53 ± 0.83	4.30 ± 0.73	4.87 ± 0.85	** < 0.001**
LDL cholesterol mmol/L	2.75 ± 0.65	2.59 ± 0.57	2.95 ± 0.70	** < 0.001**
HDL cholesterol mmol/L	1.45 ± 0.31	1.40 ± 0.31	1.53 ± 0.28	**0.001**
Triglycerides mmol/L	0.78 ± 0.54	0.73 ± 0.42	0.85 ± 0.68	0.140
Uric acid micromol/L	232 ± 60	250 ± 60	214 ± 60	** < 0.001**
Other autoimmune diseases %	29.1	25.1	34.8	0.140

Data are presented as mean and standard deviation unless otherwise specified.

*BMI-SDS* body mass index-standard deviation score, *HbA1c* glycated hemoglobin, *LDL* low density lipoprotein, *HDL* high density lipoprotein.

*Females vs males. For comparisons between groups two-tailed t-test was used.

**Mann–Whitney test.

Significant values are in bold.

### Psychometric properties of the Italian version of the EES-C questionnaire

The internal consistency of the Italian version of the EES-C questionnaire was excellent: the Cronbach's alpha coefficient was 0.934 in the whole sample, 0.944 in males and 0.925 in females. The convergent validity is confirmed by the positive and significant correlation between the EES-C score and that of the DEPS-R (r = 0.325, p < 0.001).

For the factor analysis, only components with an eigenvalue > 1 were extracted and after evaluation with Cattel's Scree test. The KMO value was 0.920 and the Bartlett test was significant. Three factors were extracted, which together explained 55% of the total variance of the items. Only items with a correlation > 0.40 were considered. The first factor was loaded by 12 items and explained 41% of the variance (eigenvalue 10.4). It represented food intake in response to unsettled and anxiety (*unsettled and anxiety subscale*, EES-C/UA). The second factor was loaded by 8 items, explained 8% of the variance (eigenvalue 1.9) and was related to symptoms of depression and frustration (*depression and frustration subscale*, EES-C/DF). Finally, the third factor was loaded by 5 items and accounted for 6% of the total variance (eigenvalue 1.4) and was correlated with symptoms of anger (*anger subscale*, EES-C/ANGER). Cronbach's coefficients for the three subscales were 0.848 for the EES-C/UA, 0.814 for the EES-C/DF and 0.925 for the EES-C/ANGER subscale. The three subscales were different from those in the original version of the EES-C questionnaire. Supplementary Table S1.

### Results of the EES-C questionnaire

The median score on the EES-C questionnaire was 31 (IQR 11, 44) in the whole sample, 28.5 (IQR 7, 44) in boys and 33.5 (IQR 17, 42) in girls, without differences. By dividing patients into age groups (10–12 years, 13–15 years, 16–17 years), 16–17 years old had a higher EES-C score than 10–12 years old (33 ± 18 vs 24 ± 18, p = 0.015).

### Results of the DEPS-R questionnaire

The mean score of the DEPS-R questionnaire score was 16 ± 11 and was higher in girls than in boys (18 ± 12 vs 14 ± 10, p = 0.007). There were no significant differences in the three age groups. In this study the internal consistency (Cronbach's alpha) of the DEPS-R questionnaire was good (0.84).

### Relationship between EES-C score and study variables

Subjects in the higher quartile of EES-C score compared to those in the lower quartile had higher HbA1c values: 6.9% (IQR 6.7–8.1%) vs 6.7% (IQR 6.2–7.0%), p = 0.007. Total cholesterol, LDL and age were also significantly higher (Table 2). In the higher quartile of EES-C scores the prevalence of subjects with DEB was 39% compared to 11% in the lower quartile (p = 0.001) (Table 2).

**Table 2:** Comparison between patients in the 1st (lower) quartile and 4th (higher) quartile of EES-C values.

Variables	EES-C 1st quartile	EES-C 4th quartile	p-value
Number	53	53	
Females N (%)	16 (30.2)	20 (37.7)	0.538
DEPS-R score	9 ± 7	18 ± 11	**0.015**
DEB N (%)	6 (11.3)	21 (39.6)	**0.001**
Age years	14.1 ± 2.2	14.9 ± 2.0	**0.036**
Diabetes duration years	6.3 ± 3.8	6.5 ± 4.0	0.804
BMI-SDS	−0.50 ± 1.0	−0.10 ± 1.0	**0.043**
**Insulin treatment %**			
Multiple daily injections	71.7	62.3	0.408
Pump	28.3	37.7	
Insulin dose U/kg/die	0.9 ± 0.2	01.0 ± 0.2	0.843
HbA1c median (IQR) %	6.7 (6.2, 7.0)	6.9 (6.7, 8.1)	0.007*
mmol/mol	50 (44, 53)	52 (50, 65)	
Total cholesterol mmol/L	4.22 ± 0.67	4.58 ± 0.85	**0.020**
LDL cholesterol mmol/L	2.54 ± 0.52	2.80 ± 0.70	**0.031**
HDL cholesterol mmol/L	1.40 ± 0.31	1.42 ± 0.26	0.483
Triglycerides mmol/L	0.67 ± 0.29	0.80 ± 0.55	0.142
Uric acid micromol/L	238 ± 59	4238 ± 59	0.976
Other autoimmune diseases %	22.6	41.5	0.061

Data are presented as mean and standard deviation unless otherwise specified.

For comparisons between groups two-tailed t-test was used.

*IQR* inter quartile range, *DEPS-R* diabetes eating problem survey-revised, *EES-C* emotional eating scale for children and adolescents, *DEB* subjects with disordered eating behaviors (DEPS-R score ≥ 20), *BMI-SDS* body mass index-standard deviation score, *HbA1c* glycated hemoglobin, *LDL* low density lipoprotein, *HDL* high density lipoprotein.

*Mann–Whitney test.

Significant values are in bold.

### Relationship between DEB and study variables

In the total sample 61 subjects had DEB (DEPS-R ≥ 20) (28.8%). The prevalence was significantly higher in girls than in males (41.9% vs 19.8% p < 0.001). Subjects with DEB had a higher EES-C test score than those without DEB: 35 (IQR 24, 46) vs 27 (IQR 6, 42) p = 0.001. This difference also persists when considering the two sexes separately: boys 37 (IQR 22, 44) vs 21 (IQR 5, 42) p = 0.041; girls 37 (IQR 24, 49) vs 32 (IQR 11, 38) p = 0.036. DEB subjects also had higher HbA1c, BMI-SDS and total cholesterol (Table 3). Dividing patients with DEB by age groups and comparing the EES-C score there were no significant differences (p = 0.38).

**Table 3:** Characteristics of patients with and without disordered eating behaviors (DEB).

Variables	DEB	NO-DEB	p-value
All N (%)	61 (28.8)	151 (71.2)	< 0.001*
Females N (%)	36 (41.9)	50 (58.1)	
Males N (%)	25 (19.8)	101 (80.2)	
EES-C score median (IQR)	37 (24.5, 46.5)	27 (6, 42)	0.001**
DEPS-R score	30 ± 7	10 ± 5	** < 0.001**
Age years	14.5 ± 1.9	14.4 ± 2.1	0.681
Diabetes duration years	6.6 ± 3.9	6.3 ± 4.1	0.710
BMI-SDS	0.30 ± 0.9	-0.50 ± 0.9	** < 0.001**
**Insulin treatment %**			
Multiple daily injections	75.4	69.5	0.491
Pump	24.6	30.5	
Insulin dose U/kg/die	1.0 ± 0.2	0.9 ± 0.2	0.155
HbA1c median (IQR) %	7.9 (7.2, 8.5)	6.7 (6.4, 7.3)	< 0.001**
mmol/mol	63 (55, 69)	50 (46, 56)	
Total cholesterol mmol/L	4.87 ± 0.93	4.40 ± 0.75	**0.001**
LDL cholesterol mmol/L	2.93 ± 0.65	2.67 ± 0.65	**0.011**
HDL cholesterol mmol/L	1.50 ± 0.28	1.42 ± 0.31	0.065
Triglycerides mmol/L	0.93 ± 0.76	0.72 ± 0.42	0.054
Uric acid micromol/L	232 ± 54	232 ± 65	0.596
Other autoimmune diseases %	34.4	27.8	0.430

Data are presented as mean and standard deviation unless otherwise specified.

For comparisons between groups two-tailed t-test was used.

*IQR* inter quartile range, *DEPS-R* diabetes eating problem survey-revised, *EES-C* emotional eating scale for children and adolescents, *DEB* subjects with disordered eating behaviors (DEPS-R score ≥ 20), *NO-DEB* subjects without disordered eating behaviors (DEPS-R score < 20), *BMI-SDS* body mass index-standard deviation score, *HbA1c* glycated hemoglobin, *LDL* low density lipoprotein, *HDL* high density lipoprotein.

*Females with DEB vs males with DEB (chi-square test).

** Mann–Whitney test.

Significant values are in bold.

Multiple linear regression showed a significant correlation between DEPS-R score and HbA1c, BMI-SDS and EES-C score in the whole group and in boys and girls separately. Supplementary Table S2.

### Relationship between DEB symptoms, emotional eating and metabolic control

In the total sample there were 49 (23.1%) patients with *disinhibition*. The EES-C score was higher than in subjects without disinhibition. BMI-SDS, HbA1c, total cholesterol and LDL-cholesterol were also higher. The number of patients with *restriction and body dissatisfaction* was 29 (13.4%). The EES-C score was not different from those of patients without the symptom. The HbA1c and BMI-SDS values were higher. Finally, *compensatory behaviors* were present in ten patients (4.7%). The EES-C score and the BMI-SDS were not different compared to subjects without the symptom. Glycated haemoglobin was higher. Comparisons between subjects with or without symptoms of DEB are presented in Table 4.

**Table 4:** Comparison between subjects with or without symptoms of eating disorders.

Variable	Disinhibition			Restriction and body dissatisfaction			Compensatory behaviors		
	Yes	No	p value	Yes	No	p value	Yes	No	p value
Number (%)	49 (23.1)	163 (76.9)		29 (13.7)	183 (86.3)		10 (4.7)	202 (95.3)	
EES-C median (IQR)	37 (26,46)	27 (6,42)	** < 0.001**	34 (19,43)	30 (8,45)	0.412	42 (32,51)	30 (10,42)	0.06
HbA1c median (IQR)	7.8 (7.1, 8.3)	6.7 (6.4, 7.4)	< 0.001*	7.8 (7.0, 8.4)	6.8 (6.5, 7.8 )	0.001*	7.8 (6.8, 10.3)	7.0 (6.5, 7.9)	0.046*
% mmol/l	62 (54, 67)	50 (46, 57)		62 (53, 68)	51 (48, 62)		62 (51, 89)	53 (48, 63)	
BMI-SDS	0.05 ± 1.05	−0.33 ± 0.96	**0.026**	0.59 ± 0.97	−0.38 ± 0.93	** < 0.001**	0.18 ± 1.07	−0.25 ± 0.99	0.851
Total cholesterol, mmol/L	4.90 ± 0.98	4.43 ± 0.75	**0.004**	4.71 ± 0.57	4.51 ± 0.85	0.088	4.35 ± 0.70	4.56 ± 0.83	0.422
LDL cholesterol, mmol/L	2.95 ± 0.73	2.67 ± 0.62	**0.018**	2.90 ± 0.52	2.72 ± 0.67	0.086	2.51 ± 0.49	2.75 ± 0.65	0.172
HDL cholesterol, mmol/L	1.50 ± 0.26	1.42 ± 0.31	0.175	1.48 ± 0.23	1.45 ± 0.31	0.520	1.50 ± 0.26	1.45 ± 0.31	0.445
Triglycerides, mmol/L	0.95 ± 0.81	0.73 ± 0.42	0.072	0.77 ± 0.29	0.78 ± 0.58	0.787	0.72 ± 0.41	0.78 ± 0.55	0.685
Uric acid, micromol/L	232 ± 48	232 ± 65	0.820	238 ± 65	232 ± 59	0.586	226 ± 30	232 ± 59	0.607

Data are expressed as mean and standard deviation unless otherwise specified. For comparisons between groups two-tailed t-test was used.

*IQR* inter quartile range, *EES-C* emotional eating scale for children and adolescents, *BMI-SDS* body mass index-standard deviation score, *HbA1c *glycated hemoglobin, *LDL* low density lipoprotein, *HDL* high density lipoprotein.

*Mann–Whitney test.

Significant values are in bold.

A total of 64 patients (30.2%) had symptoms of DEB: 41 had only one symptom (19.3%), 22 had two symptoms (10.4%) and only one patient had three symptoms (0.5%).

## Discussion

Our study shows that emotional eating is related to metabolic control (HbA1c, plasma lipids) in patients with T1D aged 10–17 years and that subjects with DEB have a higher emotional eating score than those without DEB.

To our knowledge this is the first work to specifically study emotional eating in pediatric T1D and correlate it with DEB. Wheleer et al., investigating the relationship between intuitive eating and metabolic control in a group of 38 adolescents with T1D, found a higher frequency of emotional eating than in a control group and a positive relationship with HbA1c values. However, the study, which was carried out on a small number of patients, did not analyse the relationship between emotional eating and DEB^27^.

Recent studies in adults with T1D have shown that binge episodes and voluntary reduction of insulin therapy are preceded by an intensification of negative emotional states in the periods immediately preceding them. Guilt, frustration, and stress related to the burden of diabetes are the main negative emotions reported by patients^32,33^.

In this study, emotional eating, i.e. the use of food to cope with negative emotions, was greater in older adolescents (16–17 years), but there were no significant gender differences. Subjects with higher EES-C scores had higher HbA1c, total and LDL cholesterol values. These alterations in plasma lipids could be linked to an increased intake of energy-dense foods rich in sugars and fats, as described in adolescents with emotional eating in the general population^22–24^.

Emotional eating was unrelated to the type of insulin therapy (with pump or multiple daily injections) and to associations with other autoimmune diseases.

In our sample of children and adolescents, the prevalence of DEB was 28%, the same as found in a large national Italian sample of adolescents with T1D^10^ and in agreement with literature data, the prevalence was higher in girls than in boys. Subjects with DEB had higher HbA1c, BMI-SDS, total and LDL cholesterol values than those without DEB^10–14^.

Subjects with DEB had a significantly higher EES-C score than subjects without DEB, and this finding persisted even when considering boys and girls separately. When divided by groups age, there were no significant differences in subjects with DEB comparing the EES-C scores, which may mean that the emotional eating plays the same role in early, middle, and late adolescence.

Our choice to consider positive to the items of the DEPS-R only patients who respond *very often/always* allowed us to select those who have clinically relevant symptoms. Nevertheless, 30% of the T1D adolescents in our sample had at least one symptom of DEB.

*Disinhibition* (overeating and binge eating) was the most frequent symptom of DEB in our patients with T1D (22.7%). Patients with disinhibition have a significantly higher EES-C score than those without the symptom. HbA1c and BMI-SDS were also higher. Binge eating, is the main symptom of binge eating disorder and bulimia nervosa and in both pathologies it is strongly associated with emotional eating^34,35^. Our study shows that even in children and adolescents with T1D, overeating and binge eating are associated with emotional eating. In youth with T1D, binge eating is associated with psychosocial and physiological factors related to the disease^36,37^. It can be triggered by the inability to cope with negative emotions that are neutralized by the use of food^32^. In this, a potential role can be played by diabetes-specific distress, which refers to the negative emotions arising from living with diabetes and the burden of self-management and was reported in one in three adolescents with T1D^38,39^. Dealing with emotion is one of the diabetes-specific stressors found by Chao et al. in a sample of 205 early adolescents with T1D. The others were just having diabetes and managing diabetes^40^. Dealing with emotions included uncertainty about the future and the lifelong responsibility of diabetes. This agreed with the result of the Exploratory Factor Analysis of the EES-C questionnaire that highlights the high importance of the *unsettled and anxiety* factor. In addition, binge episodes can be a consequence of food restriction with a violation of abstinence resulting in loss of control eating^41^. Finally, large glycemic fluctuations due to insulin therapy can lead to hypothalamic dysregulation of hunger and satiety cues. During hypoglycemic episodes, patients experience intense hunger with loss of control eating and overeating^37^.

*Restriction and body dissatisfaction* (diet, omission of meals, and internalization of thinness), was present in 13.4% of our children and adolescents with T1D. These patients have an EES-C score similar to those of subjects without the symptom. The values of HbA1c are instead significantly higher. Food restriction and internalization of thinness are the main symptoms of anorexia nervosa (AN). In adolescents most forms of anorexia nervosa are of the restrictive type^42,43^ without bingeing and purging, which could justify the lack of high value for emotional eating. Unexpectedly, subjects with restriction and body dissatisfaction had a higher BMI-SDS than subjects without the symptom. It can be hypothesized that in patients with T1D, subjected to dietary rules associated with insulin therapy, the dietary restriction may be only cognitive and not also associated with a calorie restriction. However, this aspect was not addressed in our study. Also, patients who restrict may be more likely to experience hypoglycemic episodes that require intake of high-calorie sugary foods. Restriction can be also strongly associated with episodes of overeating.

Finally, *compensatory behaviors* (insulin reduction/omission and self-induced vomiting), the main symptom of bulimia nervosa and anorexia nervosa subtype binging/purging, was the least frequent symptom in our sample of T1D patients (4.6%). The EES-C scores were higher than in subjects without the symptom but the difference was not significant (p = 0.06). HbA1c values are higher. In our case series, compensatory behaviors consisted almost exclusively of subjects who reduced/omitted insulin therapy while only one patient had self-induced vomiting (0.5%). Literature data agree that the omission of therapy is responsible for the worsening of metabolic control^5–7^. The reduction/omission of insulin therapy is likely to be linked to psychological processes other than emotional eating, such as denial of the disease, self-destructive and suicidal ideation, and fear of severe hypoglycemia as observed in the study of Schober^44^.

Our study shows that the Italian version of the EES-C questionnaire presented excellent internal consistency and acceptable convergent validity. The factors are different from those of the original questionnaire but this probably reflects the emotional situation of young people with type 1 diabetes. The original EES-C questionnaire was validated in 159 normal weight or overweight subjects aged 8–18 years.

The high importance of the *unsettled and anxiety* component, compared to the original EES-C questionnaire, probably reflects the difficulties faced in the daily management of diabetes. Metabolic control depends on a large number of components, including insulin therapy, ingested carbohydrates, physical activity, emotional state, and stress, which often frustrate the patient's work and commitment, causing persistent uncertainty in achieving optimal blood sugar levels^45^.

Our study has several limitations. First, it is a cross-sectional study and does not allow us to draw causal relationships between emotional eating and DEB. Second, it is monocentric so the data cannot be extended to all subjects with T1D. However, they can be considered representative of children and adolescents with diabetes in Sardinia because our centre follows more than 50% of patients in the region from all areas of the island. Finally, the questionnaire Emotional Eating Scale for Children and Adolescents, in its original version, does not have a cut-off value so it is not possible to classify subjects as suffering from emotional eating and this should be the subject of another study.

In conclusion, our study shows that emotional eating is positively correlated with HbA1c and plasma lipids in patients with T1D and that subjects with disordered eating behaviors have a higher emotional eating score than those without DEB. All symptoms of DEB (disinhibition, compensatory behaviors, restriction and body dissatisfaction) were associated with a higher emotional eating score, but only for disinhibition the effect was significant. All symptoms were also associated with higher HbA1c values. Patients with T1D and disinhibition, have also higher BMI and plasma lipids values than those without food dyscontrol. In these youth, emotional eating could be the subject of psychotherapeutic intervention with a preventive effect on DEB. Compensatory behavior and restriction and body dissatisfaction seem to be less related to emotional eating and are probably associated with different psychological processes.

## Methods

### Characteristics of participants

We studied patients with T1D aged 10–17 years followed in the Pediatric Diabetology Unit of ASL Cagliari, Italy. The subjects had been diagnosed with diabetes for at least 12 months and were autonomous in the administration of insulin therapy. Patients with a diagnosis of eating disorder already being treated or other psychiatric disorders (depression, autism, ADHD, etc.) were excluded from the study. Subjects with other chronic diseases (neurological, genetic, hematological, etc.) or an inability to understand the meaning of the questionnaires were also excluded.

### Procedure

This cross-sectional study was conducted in the period January-December 2019 with the approval of the AOU Cagliari Ethics Committee. Patients were asked, at the end of a routine visit, to complete two questionnaires: the Diabetes Eating Problems Survey-Revised (DEPS-R) and the Emotional Eating Scale for Children and Adolescents (EES-C). The questionnaires were completed after written consent was obtained from both the parents and the recruited subjects.

### Demographic and clinical data

Demographic (age, sex, duration of the disease), anthropometric (weight, height, BMI, BMI-SDS), therapeutic (type of insulin therapy, i.e., pump or multiple daily injections, daily insulin dose) and metabolic (HbA1c, total cholesterol, HDL, LDL, triglycerides, uric acid) data were taken from the patients' medical records. The presence of other autoimmune diseases associated with type 1 diabetes was also recorded: chronic thyroiditis, coeliac disease and chronic autoimmune gastritis (positivity for anti-parietal cell antibodies, APCA).

Glycated hemoglobin (HbA1c) was determined by the immunoturbidimetric method, using the Siemens DCA Vantage Analyser.

### Questionnaires

#### DEPS-R

The DEPS-R is a self-administered questionnaire with 16 items, a Likert scale *never/rarely/sometimes/often/very often/always,* and a score from 0 to 5. A higher value indicates greater pathology. The questionnaire was validated in Italian^46^. In accordance with the previous studies subjects with a score of ≥ 20 were considered to have DEB^10,47^.

Furthermore, independent of the total score and in accordance with Calcaterra's work, the DEPS-R items were used to define subjects with *disinhibition*, *compensatory behaviors, restriction and body dissatisfaction*, which represent the main phenotypes of disordered eating behaviors. Patients who responded often/very often to at least one of the 4 items were considered positive for the symptom. Each subject can have more than one symptom (Table 5).

**Table 5:** Subdivision of DEPS-R items into clinimetric factors (symptoms).

Factors (symptoms)	DEPS-R items
Disinhibition	3. Other people have told me that my eating is out of control 5. I eat more when I am alone than when I am with others 14. I feel that my eating is out of control 15. I alternate between eating very little and eating huge amounts
Compensatory behaviors	4. When I overeat, I don’t take enough insulin to cover the food 8. I make myself vomit 9. I try to keep my blood sugar high so that I will lose weight 13. After I overeat, I skip my next insulin dose
Restriction and body dissatisfaction	1. Losing weight is an important goal to me 2. I skip meals and/or snacks 11. I feel fat when I take all of my insulin 16. I would rather be thin than to have good control of my diabetes

Subjects who respond very often/always to at least one of the four items are considered to have the symptom. Each subject may also have more than one symptom.

#### EES-C

The EES-C is a self-administered 25-item questionnaire with a Likert scale *I have no desire to eat/I have a small desire to eat/I have a moderate desire to eat/I have a strong desire to eat/I have a very strong desire to eat* and a score from 0 to 4. The questionnaire examines the desire to eat in response to 25 different emotions^26^. Higher values indicate greater emotional eating. The EES-C questionnaire has three subscales: Anxiety/anger/frustration subscale, Depression subscale, and Unsettled subscale. The questionnaire was not validated in Italian so the original version was translated into Italian by a team with a diabetologist, a pediatrician and a psychologist with experience in diabetes and eating disorders. The draft of the Italian instrument was then retranslated into English by a native speaker interpreter and finally revised by the research team to evaluate any corrections. The translated items were then pretested to evaluate their comprehensibility on a small sample of 10 adolescents with type 1 diabetes followed in our center.

### Statistical analysis

The internal consistency of the EES-C questionnaire was assessed using Cronbach's alpha. Values > 0.90 indicate an excellent consistency, values between 0.70 and 0.90 indicate good consistency, values between 0.60 and 0.70 indicate acceptable consistency, values between 0.50 and 0.60 indicate poor consistency, and values < 0.50 indicate unacceptable consistency^48^. For convergent validity, the correlation with the score of the DEPS-R questionnaire was assessed using Person's correlation coefficient (r) because binge eating, the most frequent symptom of DEB in adolescents with type 1 diabetes, is correlated with emotional eating^21^.

Then, we performed an Exploratory Factor Analysis (EFA) using the principal axis factoring method with varimax rotation, the same as those used by Tanofsky in his work validating the EES-C questionnaire in children and adolescents^26^. To assess the appropriateness of the data for factor analysis, the Kaiser–Meyer–Olkin (KMO) test for measuring sample adequacy and Bartlett’s test for sphericity were used. KMO should be > 0.50 and the chi-square value of Bartlett's test should be significant^49^.

Continuous distributed variables are expressed as the mean and standard deviation, otherwise they are expressed as the median and interquartile range (IQR). Categorical variables are presented as absolute numbers and percentages. The EES-C and DEPS-R questionnaires were analysed as a total score, and then divided by gender and age groups (10–12, 13–15, 16–17 years).

Since the EES-C scale does not have a cut-off value, to evaluate the association of emotional eating with the study variables, the total sample of diabetic patients was divided into quartiles based on the EES-C score values. The first and fourth quartiles were compared to evaluate the differences between demographic (age, sex, duration of diabetic disease), anthropometric (BMI-SDS), therapeutic (type of insulin therapy, daily insulin dose), metabolic (HbA1c, total cholesterol, HDL, triglycerides, uric acid) and clinical variables (association with other autoimmune diseases).

Subsequently, subjects with DEB (DEPS-R score ≥ 20) were compared with subjects without DEB for all study variables.

To evaluate the effects of the study variables on the DEPS-R score, a linear regression analysis was performed using the DEPS-R score as a dependent variable and the EES-C score, demographic, anthropometric, therapeutic, metabolic and clinical variables as independent variables. Tolerance values > 0.1 were considered acceptable to rule out multicollinearity. Variables with skewness and/or kurtosis >| 1 | (HbA1C and EES-C score) were transformed (square root) to perform linear regression analysis and Pearson's correlation.

Finally, to evaluate the association of the three symptoms of DEB with the EES-C score and with the metabolic variables (HbA1c, total cholesterol, HDL, triglycerides, uric acid) we created three groups: the first group included all patients with disinhibition, the second group included those with restriction and body dissatisfaction, and the third group included those with compensatory behaviors. Each nonexclusive group was compared with all subjects without the corresponding symptoms for the values of the variables indicated above.

For comparisons between groups, the two-tailed t test or the Mann–Whitney test was used when indicated. For the comparison between categorical variables the chi-square test was used. One-way analysis of variance with Tukey's post hoc test was used for comparisons between age groups. Values of p < 0.05 were considered significant. Statistical analysis was performed with the SPSS 28.0 package.

### Ethical approval

The study was performed in accordance with the ethical standards as laid down in the 1964 Declaration of Helsinki and its later amendments or comparable ethical standards and was approved by the AOU Cagliari Ethics Committee.

### Informed consent

Informed consent was obtained from all study participants.

## Supplementary Information

Supplementary Tables.

## Data Availability

The datasets analyzed during the current study are available from the corresponding author on reasonable request.
