# Supplementary material for: Emotional eating and disordered eating behaviors in children and adolescents with type 1 diabetes
Source: Sci Rep. 2022 Dec 17;12:21854. doi: 10.1038/s41598-022-26271-2 (PMC9759523; doi:10.1038/s41598-022-26271-2)
Supplement: Supplementary file 1 — Supplementary Tables. [file 41598_2022_26271_MOESM1_ESM.pdf]

EMOTIONAL EATING AND DISORDERED EATING BEHAVIORS IN CHILDREN AND ADOLESCENTS WITH TYPE 1 DIABETES

Carlo Ripoli[1], Maria Rossella Ricciardi[1], Ester Zuncheddu[1], Maria Rosaria Angelo[1], Anna Paola Pinna[2], Daniela Ripoli[3]

[1] Pediatric Diabetology Unit - ASL Cagliari, Sardinia, Italy
[2] Pediatric Emergency Unit, ARNAS G. BROTZU Cagliari, Sardinia, Italy
[3] Quartu Sant'Elena (Cagliari), Sardinia, Italy

Corresponding author:
Carlo Ripoli
Pediatric Diabetology Unit
ASL Cagliari, Italy
Tel. +39 070 52963478
E-mail: carloripoli@tin.it

Supplementary table S1. Exploratory Factor Analysis of the Italian version and original version of the EES-C questionnaire.

		**Italian version of EES-C**			**Original EES-C**		
		Factor 1 *Unsettled/anxiety*	Factor 2 *Depression/frustration*	Factor 3 *Anger*	Factor 1 *Anger/anxiety/frustration*	Factor 2 *Depression*	Factor 3 *Unsettled*
1	Resentful	0.583					0.70
2	Discouraged	0.537					0.63
3	Shaky	0.590					
4	Worn out		0.461				
5	Not doing enough	0.681				0.57	
6	Excited	0.598					0.52
7	Disobedient	0.629					0.72
8	Down		0.614			0.84	
9	Stressed out		0.518			0.79	
10	Sad		0.745			0.73	
11	Uneasy	0.777			0.73		
12	Irritated			0.524	0.64		
13	Jealous	0.665			0.68		
14	Worried		0.415			0.62	
15	Frustrated		0.560		0.60		
16	Lonely		0.636			0.68	
17	Furious			0.764	0.79		
18	On edge			0.797	0.73		
19	Confused	0.752			0.74		
20	Nervous			0.707	0.68		
21	Angry			0.746	0.64		
22	Guilty	0.600			0.69		
23	Bored		0.535			0.58	
24	Helpless	0.769			0.61		
25	Upset	0.612			0.63		

In the Italian version of EES-C only items with correlation >0.40 were considered

Supplementary table S2. Linear regression analysis of variables predicting DEPS-R.

	All T1D participants (212)			T1D males (126)			T1D females (86)		
Variable	beta	p	Collinearity tolerance	beta	p	Collinearity tolerance	beta	p	Collinearity tolerance
HbA1c	0.416	<0.001	0.943	0.327	<0.001	0.948	0.318	0.002	0.972
EES-C score	0.310	<0.001	0.928	0.320	<0.001	0.958	0.256	0.009	0.990
BMI-SDS	0.148	0.027	0.984	0.271	0.003	0.988	0.451	<0.001	0.977

Only variables significantly contributing to the model are showed ($p<0.05$). EES-score and HbA1c were transformed (square root).
T1D = Type 1 Diabetes. HbA1c = Glycated hemoglobin. BMI-SDS = Body Mass Index- Standard Deviation Score
EES-C = Emotional Eating Scale for Children and Adolescents
